# Antifungal Efficacy and Surface Properties of Conventional and 3D-Printed Denture Base Materials Modified with Titanium Tetrafluoride (TiF_4_): In Vitro Study

**DOI:** 10.3390/polym17101403

**Published:** 2025-05-20

**Authors:** Zahra A. Alzaher, Fatimah A. Aldobais, Zainab Albazroun, Fatimah M. Alatiyyah, Reem Abualsaud, Haidar Alalawi, Ahmad M. Al-Thobity, Soban Q. Khan, Mohammed M. Gad

**Affiliations:** 1 Fellowship in Prosthodontics, Department of Substitutive Dental Science, College of Dentistry, Imam Abdulrahman Bin Faisal University, P.O. Box 1982, Dammam 31441, Saudi Arabia; 2 College of Dentistry, Imam Abdulrahman Bin Faisal University, P.O. Box 1982, Dammam 31441, Saudi Arabia; 2220005933@iau.edu.sa (F.A.A.); 2190003488@iau.edu.sa (Z.A.); 3 Department of Genetic Research, Institute for Research and Medical Consultations (IRMC), Imam Abdulrahman Bin Faisal University, P.O. Box 1982, Dammam 31441, Saudi Arabia; fmalatiyyah@iau.edu.sa; 4 Department of Substitutive Dental Sciences, College of Dentistry, Imam Abdulrahman Bin Faisal University, P.O. Box 1982, Dammam 31441, Saudi Arabia; rabualsaud@iau.edu.sa (R.A.); haalalawi@iau.edu.sa (H.A.); aalthobity@iau.edu.sa (A.M.A.-T.); mmjad@iau.edu.sa (M.M.G.); 5 Department of Dental Education, College of Dentistry, Imam Abdulrahman Bin Faisal University, P.O. Box 1982, Dammam 31441, Saudi Arabia; sqkhan@iau.edu.sa

**Keywords:** removable dental prosthesis, 3D printing, surface properties, color, *Candida albicans*, titanium tetrafluoride

## Abstract

**Background:** *Candida albicans* adhesion to denture base materials is a primary contributor to denture stomatitis. To address this issue, numerous studies have explored the incorporation of various additives into denture base resins to enhance their antifungal properties. Titanium tetrafluoride (TiF_4_) is an inorganic fluoride compound that has proven antimicrobial properties but has not yet been tested with denture materials. This study aimed to evaluate the effect of TiF_4_ addition into different denture base materials on antifungal activity, surface roughness, hardness, and color properties. **Methodology:** A total of 200 disc-shaped specimens were prepared—100 heat-polymerized acrylic resins and 100 3D-printed NextDent resins. Four different concentrations of TiF_4_ were incorporated: 1 wt%, 2 wt%, 3 wt%, and 4 wt% for both resins, while one group of each resin remained unmodified as a control. All specimens were subjected to thermal cycling for 5000 cycles, and four tests were conducted: *Candida albicans* adherence, surface roughness, hardness, and color change. A scanning electron microscope (SEM) was used to prove *Candida albicans* colonies’ adhesion on the specimens’ surfaces, and Fourier-transformed infrared (FTIR) analysis was performed to show the presence of TiF_4_ in the resin material; data were analyzed using one-way ANOVA followed by a post hoc test (α = 0.05). **Results:** TiF_4_ significantly reduced the *Candida albicans* adhesion to heat-polymerized specimens (*p* < 0.001). Compared to the control group, the incorporation of TiF_4_ resulted in a substantial reduction in *C. albicans* colony counts, with reductions of approximately 97.6% in 1HP, 97.2% in 2HP, 97.4% in 3HP, and complete inhibition (100%) in 4HP. However, there was no significant difference between the 3D-printed ones (*p* = 0.913). Surface roughness, hardness, and color change of heat-polymerized groups were not significantly affected by TiF_4_ (*p* > 0.05) except the color of the group treated with 4% (*p* < 0.05). For the 3D-printed groups, no significant differences were detected between the groups regarding candida count, hardness was significantly increased at 2% TiF_4_ compared to the control (*p* = 0.002), and roughness was least with 4% TiF_4_, while the color varied significantly between the groups (*p* < 0.001). **Conclusions:** TiF_4_ addition decreased *Candida albicans* adhesion to heat-polymerized denture base materials but showed no antifungal effect on the 3D-printed resin. While roughness remained low in 3D-printed groups at higher concentrations. Hardness was not significantly altered in the heat-polymerized resin, whereas it increased significantly in the modified 3D-printed resin. Color stability was compromised at higher TiF_4_ concentrations, particularly in the 3D-printed groups. The type of denture base material and TiF_4_ concentration both influenced antifungal activity and denture surface properties.

## 1. Introduction

Poly Methyl Methacrylate (PMMA) is the most widely used denture base material due to its high mechanical and cosmetic properties [1,2]. However, it has multiple disadvantages, including polymerization shrinkage, lack of radio-opacity, possible human allergic reaction, dropping of the mechanical properties over time, low wear resistance, and porosity [3,4]. PMMA’s surface, exhibiting apparent porosity and surface roughness, can act as a reservoir for microorganisms. Furthermore, poor denture hygiene leads to biofilm formation, debris accumulation, and surface scratches, all of which result in denture stomatitis [1,2]. To overcome these drawbacks of a conventionally fabricated denture base, different modalities were suggested through introducing new resin materials and technologies to fabricate dentures, as well as modifying the available resin materials.

Recent improvements in technology included digital denture base fabrication with computer-aided design/computer-aided manufacturing (CAD/CAM), and milled and three-dimensional (3D) printing [5,6,7,8]. The 3D printing, also known as additive manufacturing, is increasingly applied in dentistry for its ability to fabricate customized and intricate structures with high precision. The process involves layer-by-layer construction from digital models, offering efficient and accurate replication of dental components. This technology has demonstrated significant potential in improving the design and production of dental appliances through both powder- and liquid-based printing techniques [9]. Compared to conventional PMMA dentures, digitally fabricated dentures have better tissue adaptation and require a fewer number of appointments [10,11]. The 3D-printed resins were found to have more surface roughness compared to heat-polymerized acrylic resins, while flexural strength, impact strength, and surface hardness are lower than heat-polymerized counterparts, as reported by Gad et al. [12].

PMMA surfaces are prone to microbial colonization, particularly by *Candida albicans* (*C. albicans*), due to their porosity and tendency to retain biofilm [13,14]. This fungal colonization is a key factor in the development of denture stomatitis, an inflammatory condition that affects 11–67% of geriatric denture wearers [15]. The ability of *C. albicans* to develop structured, drug-resistant biofilms plays a critical role in disease progression [13], contributing to recurrence rates of 30–75% even after antifungal therapy [16]. The formation of denture plaque creates a favorable environment for fungal adherence and persistence, complicating both prevention and treatment efforts [13,14]. Traditional management approaches include topical antifungal treatments such as nystatin, sometimes incorporated into soft liners for localized delivery [16]. However, these methods often provide only temporary relief and may be limited by factors such as drug leaching, reduced efficacy over time, and patient non-compliance [16,17]. Additionally, routine hygiene practices may not completely eliminate yeast contamination, especially in elderly populations where denture hygiene can be challenging [17]. Earlier strategies that incorporated antifungals like nystatin and amphotericin B into denture base materials demonstrated some effectiveness but also raised concerns about potential impacts on material integrity and the depletion of active agents over time [18]. As a result, recent research has shifted toward the integration of natural and inorganic antifungal compounds with sustained activity. Agents such as henna [19], thymoquinone [20], and neem powder [21] have shown promising antifungal effects against *C. albicans* when incorporated into denture acrylic resins, offering possible protection of the denture bases.

The denture base should have a smooth surface to decrease the chance of plaque accumulation and denture discoloration. Material hardness is known as the material’s ability to withstand permanent indentation and penetration [22]. Denture base surface properties are affected by denture hardness [23]. Rough or low hardness denture base material is susceptible to plaque accumulation and damage during finishing [2]. In addition, denture roughness has an important role in staining and plaque and bacterial accumulation, which affect the patient’s satisfaction as well as denture esthetic appearance [24,25,26,27]. Another reason for unesthetic denture appearance is color change [28]. The color of the denture base should not change during processing or actual use of the denture [29]. Color change may indicate aging or material damage that requires denture replacement [30].

Some authors found that the use of fluoride dentifrices on denture bases is effective against *C. albicans*, Staphylococcus aureus, and Pseudomonas aeruginosa [31]. Titanium tetrafluoride (TiF_4_) is an inorganic fluoride compound [32] that was proven to prevent enamel demineralization [33,34] and tooth erosion [35]. It was also successful in increasing the bond strength of some types of resin [36] and between fiber post and resin cement [37]. Regarding the antibacterial activity of TiF_4_, a study found it ineffective as an antimicrobial agent [38], while other studies proved its effectiveness at a concentration of 2% against *Streptococcus mutans* and *Lactobacillus casei* [39]. Different concentrations of TiF_4_ from 1 wt% to 4 wt% were tested in biodentine material to analyze the change in surface properties and antimicrobial effects [37]. The highest surface microhardness was found with 2 wt%, while the antimicrobial activity was significant with both 1 wt% and 2 wt% against *Enterococcus faecalis* [37]. Eskandarian et al. [40] concluded that the bactericidal activity of 12.5% TiF_4_ against *Streptococcus mutans* is significant and comparable to chlorohexidine and sodium fluoride.

To the authors’ knowledge, no previous studies have tested the effect of TiF_4_ addition to denture bases, and only one study [41] evaluated the antifungal activity of TiF_4_. Thus, the current study aimed to evaluate the effect of TiF_4_ addition to conventional heat-polymerized and recently introduced digital denture base materials in terms of antifungal activity, roughness, hardness, and color properties. The null hypothesis was that the number of *C. albicans* colonies on the denture base material and denture roughness, hardness, and color would not be affected by TiF_4_ addition.

## 2. Materials and Methods

### 2.1. Sample Size Calculation

Power analysis was performed for the purpose of sample size calculation. To serve the purpose, the parameters required were effect size (0.5), sample power (0.9), and alpha (0.05). Moreover, means and standard deviation (SD) were obtained from previously published studies [12,21]. Hence, the calculated sample size was 10 samples per group. The total number needed was 100 specimens for each material, and the number was duplicated to accommodate the candida test and surface properties tests (n = 20 per test group, 10 for candida and 10 for surface properties).

### 2.2. Specimens Grouping

Two denture base resins were used in the present study: heat-polymerized (“HP”, Probase Hot, Ivoclar Vivadent, Lichtenstein) and 3D-printed NextDent (“ND”, NextDent Denture 3D+, 3D systems, Vertex Dental B.V., Soesterberg, The Netherlands). They were used to fabricate a total of 200 discs (15 × 2 mm) specimens. Four concentrations of TiF_4_ powder (Aldrich Chemical Company, Milwaukee, WI, USA) were used: 1 wt%, 2 wt%, 3 wt%, and 4 wt% to modify the resins. The groups were divided according to the incorporated TiF_4_ concentration, with a control group that was free of any TiF_4_ content. The groups’ abbreviations are HP for heat-polymerized specimens and ND for 3D-printed specimens, along with the concentration of the TiF_4_. This nomenclature resulted in 5 groups for HP resin (HP, 1HP, 2HP, 3HP, and 4HP) and 5 groups for ND resin (ND, 1ND, 2ND, 3ND, and 4ND), where the number before each abbreviation stands for the TiF_4_ concentration. The specifications of the used materials are listed in Table 1.

### 2.3. Resin/TiF_4_ Mixture Preparation

#### 2.3.1. HP/TiF_4_ Mixture

A digital scale (Analytical Balance, XP-3000, Denver Instrument, Bohemia, NY, USA) was used to measure the TiF_4_ ratio to resin powder by weight. The TiF_4_ powder was manually mixed with the heat-polymerized acrylic resin powder for 5 min to ensure the distribution of the TiF_4_ into the resin powder. The modified powder was kept in glass containers and labeled as HP, 1HP, 2HP, 3HP, and 4HP based on the TiF_4_ concentration.

#### 2.3.2. Three-Dimensional-Printed/TiF_4_ Mixture

The 3D-printed fluid resin container was rolled in NextDent LC-3D Mixer (Vertex-Dental B.V., Soesterberg, The Netherlands) for 1 h according to the manufacturer’s recommendation to assure homogenous distribution of resin ingredients before TiF_4_ addition. A digital scale was used to measure the TiF_4_ ratio to fluid resin by weight, followed by vigorous hand mixing, then stirring for 30 min for homogenization of TiF_4_ within the resin before dispensing into the tank for the process of 3D printing [42].

### 2.4. Three-Dimensional-Printed Resin Specimens’ Preparation

Digital specimens were designed as disc shape (15 × 2 mm) using an open-source CAD software program (123D design, Autodesk, version 2.2.14, San Francisco, CA, USA). The specimen’s design was saved as Standard Tessellation Language (STL) files to be exported to a 3D-printing software. A commercially available 3D-printed NextDent resin was used to fabricate a total of 100 specimens with its 3D printer (NextDent; 5100; 3D systems, Vertex Dental B.V., Soesterberg, The Netherlands).

The specimens were printed according to the manufacturer’s recommendations with 30-degree printing orientation, and 50 µm printing layer thickness. After printing, the standardization of cleaning the specimens was performed using alcohol bath (Isopropyl Alcohol 99.9%, Saudi Pharmaceutical Industries, Riyadh, Saudi Arabia) followed by post-curing process using UV-light polymerization unit (LC-D Print Box, 3D systems, Vertex Dental B.V., Soesterberg, The Netherland) for 10 min while the specimens were submerged in a bowl filled with glycerol [43]. After post-curing, the support parts were removed by low-speed rotary instruments, and specimens were kept in water until the polishing process.

### 2.5. HP Specimens’ Preparation

HP specimens were prepared to stimulate the denture bases. To standardize the specimens’ dimensions, an additional 100 3D-resin specimen discs were printed using the previously designed disc for the printed group to replace the wax specimens. The printed discs were invested in dental stone (Fujirock EP; GC Corporation, Tokyo, Japan) inside flasks (61B Two Flask Compress; Handler Manufacturing, Westfield, NJ, USA) [44]. After flasking, discs were removed, and a separating medium (162-800-00; Vandex Isoliermittel GmbH, Hamburg, Germany) was applied to all stone surfaces. The resin (pure and TiF_4_-modified) powder was mixed with the acrylic resin liquid, according to the manufacturer’s instructions. The mixture was packed when it reached a dough stage using a pneumatic press and left aside for 30 min. Flasks were immersed in water inside a thermal curing unit (KaVo Elektrotechnisches Werk GmbH, Biberach, Germany) and processed at 74 °C for 8 h, followed by 100 °C for 1 h for polymerization. After processing, flasks were left aside for 30 min to cool down before they were opened, and HP acrylic resin specimens were retrieved. The specimens’ borders were finished using a straight handpiece with carbide acrylic bur and kept in water until the polishing process.

### 2.6. Specimens Polishing

The specimens were prepared and polished by one operator only. The prepared disc specimens of both resins were polished with 600-grit silicon carbide paper on a rotating polishing machine (Metaserv 250 Grinder-Polisher, Buehler Ltd., Lake Bluff, IL, USA) and rinsed with water. All specimens were stored in water at 37 °C for 48 h.

### 2.7. Thermal Aging

All specimens were thermally cycled using a thermal cycling machine (Thermocycler THE-1100—SD Mechatronik GmbH, Feldkirchen-Westerham, Germany), where they were subjected to 5000 cycles between 5 °C and 55 °C with a 1 min dwell time simulating 6 months of clinical use [45].

### 2.8. Fourier-Transform Infrared (FTIR) Analysis

Functional groups, it is a key instrument for investigating the bonding interactions between TiF_4_ and the resin matrix. The modifications to the structural bonding caused by the addition of TiF_4_ can be detected with the use of FTIR. The specimens were put inside an FTIR spectrometer equipped with transmission spectroscopy for FTIR. FTIR spectra covering a wavenumber range of 4000 to 400 cm^−1^ were acquired at a resolution of 4 cm^−1^, which was selected to ensure a comprehensive analysis of all potential functional groups present. Identification of bond types and any matrix chemical alterations caused by the presence of TiF_4_ becomes attainable by the characteristic peaks in the resulting spectra that correspond to specific functional groups [46].

### 2.9. Specimen Testing

#### 2.9.1. *C. albicans* Adherence Test (Colony Forming Unit, (CFU/mL))

Specimens (n = 10 per group) were placed in an ultrasonic cleaner with 70% alcohol for 15 min, then washed with distilled water 3 times to ensure cleaning, and then dried by air. Specimens were placed in artificial saliva containing 0.5 Macfarland of *C. albicans* cells (ATCC 14053) and incubated for 48 h at 37 °C inside an incubator (HF151UV CO_2_ incubator, Heal Force, Shanghai Lishen Scientific Equipment Co., Ltd., Shanghai, China). After that, the specimens were washed with phosphate-buffered saline (PBS) to remove non-adherent cells. The specimens were then kept in sterile tubes with PBS and vortexed to remove the adherent *C. albicans* cells to be cultured [21]. The direct culture method was used, and the *C. albicans* cells were measured by colony-forming unit (CFU). A 100 µL sample was taken from each previously vortexed tube, spread onto a Petri dish, and placed in an incubator for 24 h at 37 °C [21]. Then, the number of *C. albicans* colonies with clear acceptable growth was counted in each quadrant using a colony counter pen (SP Scienceware, Bel-Art Products Inc., Pequannock, NJ, USA).

A scanning electron microscope (SEM) (JEOL JSM-6510LV, JEOL Ltd., Tokyo, Japan) was used to analyze the incubated specimens. The specimens were unvortexed to keep the *C. albicans* on the surface. The samples were gold-coated using a gold-coating machine (Cressington 108 Auto Sputter Coater, Ted Pella Inc., Redding, CA, USA) and then analyzed under different magnifications for candidal adhesion.

#### 2.9.2. Surface Roughness (µM) Test

Surface roughness parameter *Ra* (averaged all peaks and valleys of the roughness profile) was measured using a noncontact optical interferometric profilometer (Contour Gt-K1 optical profiler; Bruker Nano, Inc., Tucson, AZ, USA). The machine was placed on a vibration-isolated table in a noiseless room. Each specimen was positioned horizontally under a standard camera at a selected magnification, and the area was scanned. Three scans for the surface of each specimen were taken radially (across each specimen), and the average was calculated per specimen. A software package (Vision 64; Bruker) was used to determine pit characteristics of the images, then the average surface roughness value was calculated for each group (μm) (n = 10 per group) [45].

#### 2.9.3. Hardness (VHN) Test

Specimens’ hardness was evaluated using Vickers hardness (VH) by a hardness tester (Wilson Hardness; ITW Test & Measurement, GmbH, Shanghai, China). Specimens (n = 10 per group) were immersed in distilled water, then placed horizontally on the device table under a 25 g load for 30 s [46], and the load was applied using a diamond tip that was pressed down firmly and quickly. The diagonals of the resulting indentations were measured under a microscope to determine the hardness. Each specimen underwent a total of 3 indentations made at different locations, and the average value for each specimen was calculated (VHN).

#### 2.9.4. Color Change (ΔE_00_) Test

**Figure 1:**
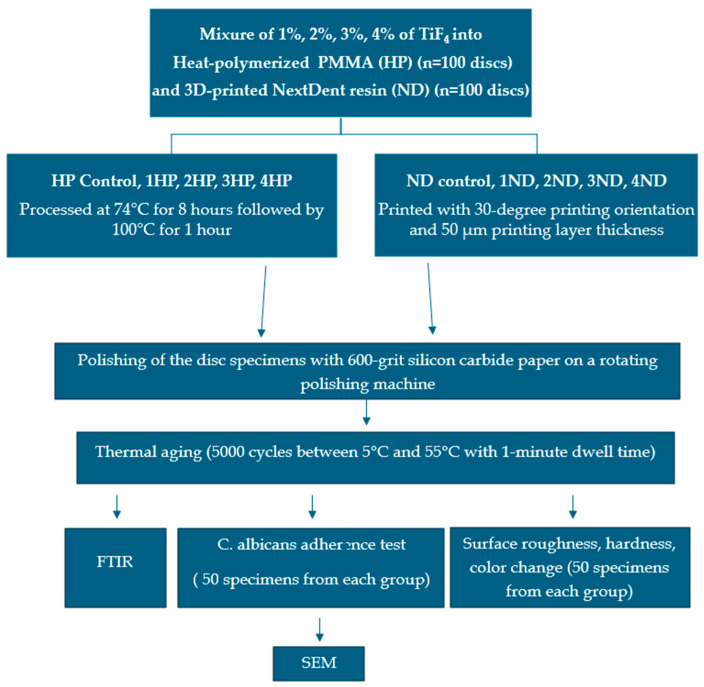
The study flow chart.

A spectrophotometer (Color-Eye^®^ 7000A Spectrophotometer, X-Rite Inc., Grand Rapids, MI, USA) was used to measure the reflectance values. The spectrophotometer was calibrated using the provided white tile and black trap per the manufacturer’s instructions. Each specimen was placed against the port, supported at the back by black reference material, and then the support arm was closed. Color measurements of the coordinates (L*, a*, b*) of the Commission Internationale de l’Eclairage (CIE) system were made for every disc against the background. The value of ΔE_00_ was calculated as detailed in previous studies [47,48]. The data were entered, and the color difference between the groups (control vs. test group) was compared [47]. The values of ΔE_00_ were evaluated to describe the perceptibility and acceptability of the color change, as prescribed in previous studies [48]. The flow chart of the study is presented in Figure 1.

### 2.10. Statistical Analysis

Data were analyzed using IBM SPSS Statistical software for Windows version 19.0 (IBM Corp., Armonk, NY, USA). To check the normality of the data, Shapiro–Wilk test was used, and insignificant results from the test provided that the data were normally distributed. Hence, parametric tests were used for the inferential analysis. The differences in live *C. albicans* colony numbers for different TiF_4_ concentrations, surface roughness, hardness, and color change were compared. The analysis of variance (one-way ANOVA) test and post hoc Tukey’s honestly significant difference test were performed to compare the differences with a *p* ≤ 0.05 significance level.

## 3. Results

### 3.1. C. albicans Adherence (CFU/mL)

The values of the CFU are presented in Table 2 and Table 3. For the HP groups, the addition of TiF_4_ significantly decreased the *C. albicans* adhesion compared to the control group (*p* = 0.000 for 1HP, 2HP, 3HP, 4HP). Between the HP-modified groups, there was no statistically significant difference with different concentrations of TiF_4_. Moreover, no candidal adhesion was observed in 4HP. For the ND group, the addition of TiF_4_ had no statistically significant effect on candidal adhesion of any group compared to the control (*p* = 0.913). The highest amount of candida adhesion was found with 4% TiF_4_ (2817.8 CFU), followed by 1%, the control group, 3%, and 2%, respectively.

**Figure 2:**
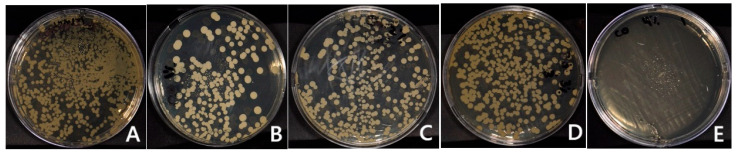
Direct culture method based on different TiF_4_ concentrations of heat-polymerized acrylic resin specimens: (**A**) Control; (**B**) 1% TiF_4_; (**C**) 2% TiF_4_; (**D**) 3% TiF_4_; (**E**) 4% TiF_4_.

**Figure 3:**
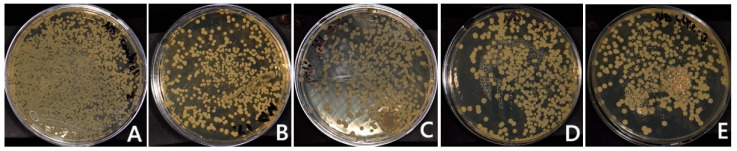
Direct culture method based on different TiF4 concentrations of 3D-printed resin: (**A**) Control; (**B**) 1% TiF_4_; (**C**) 2% TiF_4_; (**D**) 3% TiF_4_; (**E**) 4% TiF_4_.

**Figure 4:**
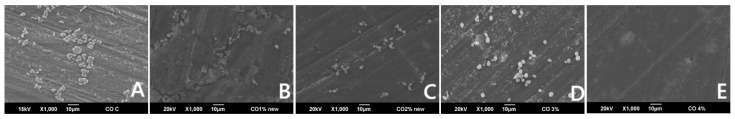
Scanning electron microscopy images of heat-polymerized acrylic resin: (**A**) Control; (**B**) 1% TiF_4_; (**C**) 2% TiF_4_; (**D**) 3% TiF_4_; (**E**) 4% TiF_4_ at the same magnification.

**Figure 5:**
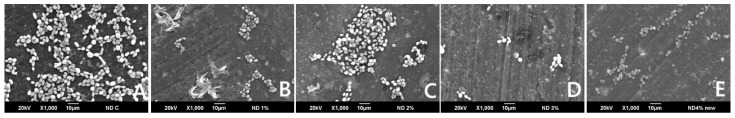
Scanning electron microscopy images of 3D-printed resin: (**A**) Control; (**B**) 1% TiF_4_; (**C**) 2% TiF_4_; (**D**) 3% TiF_4_; (**E**) 4% TiF_4_ at the same magnification.

Candida adhesion under SEM and the culture method of the groups is presented in Figure 2, Figure 3, Figure 4 and Figure 5. For the HP control group, the SEM image showed candida adhesion on a textured resin surface with parallel grooves, where yeast cells appeared as clusters in oval structures at an early colonization stage. The candida cells in 1HP are more sparsely distributed, with some clustering in crevices. For 2HP, the resin surface had fine parallel grooves, similar to the HP control but smoother than 1HP. Compared to the HP control and 1HP, fewer yeast cells were present, and they appeared more dispersed along the grooves rather than clustered in crevices. 3HP showed that, compared to previous groups, candida adhesion appeared even more reduced, with yeast cells more widely scattered and fewer clusters forming. 4HP presented a smoother resin surface with faint grooves and minimal surface irregularities, in contrast to the rougher or more textured surfaces in previous groups. Unlike the other HP groups, candida adhesion is almost absent.

The SEM images of the ND group demonstrated varying candida adhesion levels on resin surfaces with different roughness levels. The ND (control) image showed extensive candida colonization, with densely clustered yeast cells. In 1ND, adhesion is reduced, with sparser clusters and some debris present. 2ND revealed a rough resin surface with more aggregated yeast cells than 1ND, but still less extensive than the ND control. 3ND exhibited further reduction in candida attachment, with sparser clusters and smaller yeast groups. The 4ND SEM image showed a resin surface with visible grooves but minimal adhesion, where yeast cells appeared widely scattered with fewer clusters.

### 3.2. FTIR

**Figure 6:**
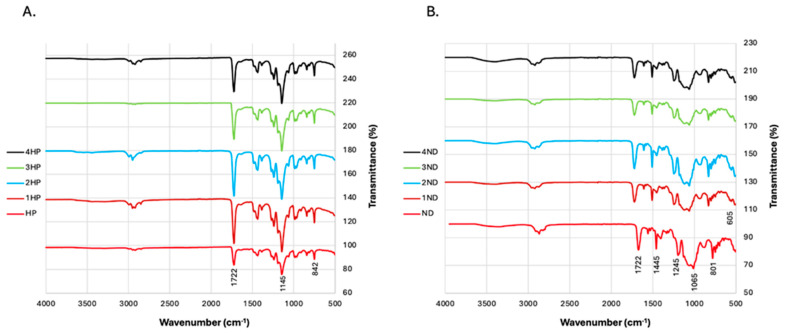
FTIR analysis of the study groups: (**A**) Heat-polymerized groups with and without TiF_4_ incorporation; (**B**) 3D-printed groups with and without TiF_4_ incorporation.

Heat-polymerized and 3D-printed resins differed chemically, as demonstrated by FTIR investigation (Figure 6). The strength of the carbonyl double bond (C=O) stretching peak, which is usually seen at 1720–1730 cm^−1^, is one of the most obvious similarities; however, with HP groups, it is often a sharper and more distinct peak. Also, the ester group, which is represented by C–O stretching, was found in 1145 and 1245 cm^−1^ in the HP and 3D-printed resins, respectively. In the FTIR analysis of TiF_4_, it is expected that Ti–F bond to produce absorption bands in the lower wavenumber region, specifically between 500 and 700 cm^−1^. In the group study, it was evident in the modified ND groups at 605 cm^−1^. Whereas in HP, it was not very obvious due to these bands being extremely weak or masked by stronger nearby absorptions from other functional groups.

### 3.3. Surface Roughness

The roughness is presented in Table 2 and Table 3. For the HP group, no significant difference was found between the modified groups and the control group (*p* = 0.062). 1HP and 3HP had higher roughness than the control group; however, they were not significantly different, while 4HP was similar in the roughness value to the control group.

For ND groups, modified groups with 1% and 2% had significantly higher roughness than the control (*p* = 0.024, 0.002, respectively). The lowest and highest roughness values were detected with 4% and 2% TiF_4_, respectively. There was a statistically significant difference between the following groups: 1ND/4ND (*p* = 0.000), 2ND/3ND (*p* = 0.009), 2ND/4ND (*p* = 0.000), and between 3ND/4ND (*p* = 0.031).

### 3.4. Hardness (VHN)

Hardness values for the HP groups ranged between 27.5 and 29.5 VHN with no significant differences between the groups (*p* = 0.169) (Table 2). The addition of TiF_4_ significantly increased the hardness of the 2ND group compared with all the other ND groups (*p* = 0.001, 0.04, 0.013, 0.019 for ND control, 1ND, 3ND, 4ND, respectively). The hardness of the ND groups ranged between 24.2 and 34.9 VHN (Table 3). 2ND had the highest significant hardness among all the groups.

### 3.5. Color Change (ΔE_00_)

The incorporation of TiF_4_ did not result in a significant color change among the HP groups (*p* > 0.05), except for 4HP, which exhibited a statistically significant difference compared to all other HP groups (*p* = 0.000, 0.000, 0.004 for 1HP, 2HP, 3HP, respectively) (Table 2). The ΔE_00_ values for all HP groups indicated that the color changes were perceptible and exceeded the threshold of clinical acceptability [48]. For the ND groups, significant differences in color change were observed across the various TiF_4_ concentrations (*p* < 0.05). The 1ND group showed a significantly greater color change than the 2ND group (*p* = 0.000), but less than the 4ND group (*p* = 0.000). The 2ND group exhibited the lowest degree of color change overall, with significantly lower ΔE_00_ values compared to all other ND groups (*p* = 0.000). The 3ND group demonstrated a significantly higher color change than the 2ND group (*p* = 0.000), but significantly less than the 4ND group (*p* = 0.000). The 4ND group recorded the highest ΔE_00_ value, indicating the most pronounced color change, significantly exceeding all other ND groups (Table 3). Similar to the HP groups, all values of the ND groups were perceptible and clinically unacceptable [48].

## 4. Discussion

This is the first study that evaluated the effect of TiF_4_ addition on the microbial adhesion, surface roughness, hardness, and color properties of heat-polymerized and 3D-printed denture base resins. Based on the findings of the present study, it was revealed that the addition of TiF_4_ significantly affected some tested properties of the resins, while others were not affected; therefore, the null hypothesis was partially accepted.

The oral cavity is subjected to temperature variations ranging from 4 °C to 60 °C, which are often simulated in vitro through thermal cycling processes [49,50]. Thermal cycling mimics the mechanical fatigue that materials experience under moist oral conditions [51]. Hence, all specimens were subjected to thermal cycling to simulate the oral environment.

TiF_4_ has been studied in dental research since 1972 [52] for various applications, including the prevention of dental caries, sealing of dentinal tubules in root canals, reducing tooth erosion, alleviating dentinal hypersensitivity, and exhibiting antibacterial properties [53]. Previous studies have shown that incorporating TiF_4_ (at concentrations of 1% and 2% by weight) into biodentine enhanced its physicochemical properties and antibacterial activity [53]. On the tooth surface, the antimicrobial effect is attributed to titanium’s interaction with phosphate in dental apatite, which forms a protective, acid-resistant “glaze” on the surface [52]. TiF_4_ has also been shown to have bactericidal effects against *Streptococcus mutans*, comparable to the efficacy of chlorhexidine [40]. In 2015, Bridi et al. attributed the TiF_4_ protective role to two primary factors: its ability to reduce surface solubility by enhancing fluoride content and the formation of a protective glaze that resists acid penetration [54].

However, no research has fully elucidated the mechanism by which TiF_4_ exerts its antimicrobial effects [55]. The current study found that a high concentration (4%) of TiF_4_ was particularly effective against *C. albicans* in the HP group. Sen et al. conducted the only study specifically addressing the antifungal properties of TiF_4_ against *C. albicans* [41]. That research found that TiF_4_ exhibited significant antifungal activity, being more effective than NaF but less potent than ethylenediaminetetraacetic acid (EDTA). The study suggested that TiF_4_ could be used as an alternative irrigating solution for root canal treatments, particularly in cases involving *C. albicans* infections [41]. Earlier studies have suggested possible mechanisms for this fluoride compound’s effect. When TiF_4_ interacts with dental enamel, titanium breaks its bond with fluoride and binds with oxygen on the surface, forming a titanium dioxide (TiO_2_) outer coat layer [56]. This titanium-rich layer, which consists primarily of TiO_2_, is more durable and acid-resistant than other fluoride agents [52], thereby reducing solubility and porosity [57]. Although the TiO_2_ layer may not be uniform due to surface porosity and irregularities, it still provides antimicrobial benefits [58]. The stable TiO_2_ layer protects against acidic bacterial activity [59]. Additionally, TiF_4_ reduced the levels of *Streptococcus sobrinus* [60] and demonstrated antibacterial effectiveness when combined with biodentine and mineral trioxide aggregate (MTA) against *Enterococcus faecalis* [53,61]. Among the TiF_4_ concentrations tested, MTA with 3% TiF_4_ exhibited the highest antimicrobial efficacy compared to 1% and 2% concentrations [61]. Similar to the results of the current study where higher concentrations showed fewer CFU in the HP group. It was proved that TiF_4_ solutions were acidic (with pH values below 2.0), and the acidity increased as the concentration of TiF_4_ rose. The acidic pH results from the hydrolysis of TiF_4_, which releases TiO_2_ and fluoride. The fluoride ions released interact with the oral environment and water, releasing protons (H+) and lowering the pH of the solution [62,63]. Hove et al. observed that higher concentrations of TiF_4_ and lower pH values enhanced the anti-erosive effect on the enamel surface [64]. The glaze-like layer produced by TiF_4_ was expected to alter microorganism adhesion, reducing biofilm growth and viability [38]. However, previous studies have only focused on dental structures and restorative materials, and no research has explored the combination of TiF_4_ with denture materials, as the present study does, to investigate its antifungal properties. The reduction in fungal growth observed could be attributed to the glazed, acid-resistant surface created by TiF_4_, which reduced fungal adhesion as well as activity on modified denture bases of the HP group.

In this study, SEM images demonstrated that surface roughness and texture influenced the extent of candida colonization, with smoother surfaces exhibiting reduced adhesion. Since TiF_4_ created a protective barrier that interfered with microbial attachment, its application on resin surfaces could further inhibit candida adhesion and biofilm development. This observation aligns with the trend seen in the SEM images, where smoother resin surfaces (such as 4HP) showed significantly lower microbial colonization. The mechanism behind this effect may be similar to TiF_4_’s ability to alter surface free energy, creating conditions that are less favorable for microbial adhesion.

In this study, TiF_4_ powder was directly mixed with either 3D-printed resin or conventional PMMA powder. Achieving a homogeneous mixture with the printed resin was challenging, as the TiF_4_ powder tended to cluster during mixing, which might lead to uneven distribution. In contrast, mixing TiF_4_ with PMMA powder resulted in a stable mixture with uniform distribution. Although the containers of the modified printed resins were placed on a roller/tilting mixer before the printing process to ensure proper TiF_4_ particle distribution, there may have been a chemical interaction between the TiF_4_ particles and the liquid resin during mixing. This interaction could have caused the particles to cluster in specific areas, preventing homogeneous distribution and leading to different antifungal results than the heat-polymerized groups. The authors suggest modifying the preparation of the printed specimens in future research by creating a TiF_4_ solution from the powder before mixing it with the resin liquid to compare the results with the current study.

Previous studies have used various methods to incorporate TiF_4_ into different agents. For instance, Pomarico et al. [65] and Bridi et al. [54] prepared a 4% TiF_4_ solution by dissolving 3.4 g of TiF_4_ in 100 mL of deionized water. Elsaka et al. [53] added TiF_4_ powder to MTA and blended it using an amalgamator to achieve a homogeneous mixture, which was then mixed with distilled water. Another approach involved creating aqueous solutions or self-etching primers by mixing TiF_4_ powder with ultrapure water or primer under constant agitation [39]. These methods could be explored in future studies with printed resins to compare them with the current findings.

Comparing the study findings on hardness and roughness with previous studies is somewhat challenging due to the limited amount of research specifically focused on the interaction between TiF_4_ and dental materials. For instance, Topaloglu et al. [66] reported that the application of TiF_4_ did not result in any significant changes in surface roughness of glass ionomer cement across other tested materials. This suggests that TiF_4_, despite its low pH and potential to interact with the surface layer, does not compromise the surface texture of the material, maintaining its structural integrity in terms of roughness. In contrast, another study found that applying TiF_4_ to commercially pure titanium surfaces increased surface roughness, particularly at higher concentrations and longer application times [67]. However, the differences in material composition and treatment protocols make direct comparison complex. This study extends this line of research by evaluating the unique interaction of TiF_4_ with heat-polymerized and 3D-printed denture base materials, offering new insights into its potential applications in prosthodontics.

The current study demonstrates that the effect of TiF_4_ on surface roughness varies depending on the material type and concentration used. In the HP group, 2HP showed a reduction in surface roughness, whereas 1HP and 3HP exhibited increased roughness compared to the control. However, 4HP presented a roughness value comparable to the control. These findings suggest that TiF4 does not consistently induce significant surface alterations, as no statistically significant differences were observed among the groups. In the ND group, roughness increased significantly at 1% and 2% TiF_4_, while 4% TiF_4_ resulted in the smoothest surface. These findings indicate that TiF_4_’s influence on surface roughness is concentration-dependent. This partially aligns with previous research, which reported no significant alterations in roughness following TiF_4_ treatment, but the present results suggest that certain conditions, such as material composition and concentration, may influence surface properties. This study shows a link between surface roughness and candida adhesion, as the rougher the surface, the more candida tends to adhere, while smoother surfaces make it harder for the yeast to attach. In the HP group, 4HP, with a roughness level similar to the control (less than 1% and 3%), had an absence of candida adhesion. Interestingly, although the 2HP group exhibited the lowest surface roughness among the HP subgroups, it did not demonstrate the least candida growth. This suggests that the tested TiF_4_ material might possess inherent antimicrobial properties, independent of surface roughness. Supporting this, the 4HP group showed higher roughness than 2HP, yet exhibited no candida adhesion. In the ND groups, 4% TiF_4_ created the smoothest surface, but the highest amount of candida growth. However, the increase in CFU among the ND groups was not statistically significant, and the surface roughness differences in the HP groups were also not significant.

While TiF_4_ has been extensively studied for its effects on enamel and dentin hardness, research on its impact on other dental materials is limited. Elsaka et al. reported that incorporating 2% TiF_4_ into biodentine significantly enhanced its physicochemical properties, including microhardness [37]. This finding is consistent with the results of the current study in the ND groups, where 2% TiF_4_ led to significant improvement in surface hardness compared to all other ND groups, suggesting that 2% TiF_4_ may be an optimal concentration for enhancing the hardness properties. However, the lack of significant differences in the HP groups suggests that the impact of TiF_4_ might depend on the type of material or its formulation. Overall, the study findings highlight TiF_4_’s potential to enhance the hardness of 3D-printed resin, and the material did not have a negative effect on the hardness of the heat-polymerized group, which favors the future usage of this material in the prosthodontic field. However, more research is needed to understand its effects on different materials.

TiF_4_ has been primarily studied for its effects on enamel and dentin, with limited research on its impact on the color change of other dental materials. A study by Nascimento et al. in 2024 [68] investigated the combination of TiF_4_ with hydrogen peroxide as a bleaching agent on bovine enamel. The study found that incorporating 1% TiF_4_ into the material did not interfere with the enamel bleaching effect. This means that TiF_4_ could be added safely without affecting the color of the original treated surface. However, specific data on TiF_4_’s direct effect on color changes of dental materials other than enamel are scarce, making comparisons with previous literature challenging. In the present study, the addition of TiF_4_ to the heat-polymerized resin did not result in significant color changes between groups (*p* > 0.05), except for the 4HP, which exhibited a significantly higher ΔE_00_ value (*p* < 0.05). On the other hand, all printed groups showed perceptible and unacceptable color changes, with significant differences observed among concentrations (*p* < 0.05). The 2ND group demonstrated the least color change, while the 4ND group showed the highest. This discrepancy may be attributed to the method of TiF_4_ incorporation; the powder was likely more uniformly dispersed within the HP resin mixture, whereas in the ND printed resin, inability to homogenize the added material by the mixing process and localized accumulation may have resulted in uneven coloration and greater variability in color among the groups, as all showed statistically significant differences from one another (*p* < 0.05). Despite the even distribution of TiF_4_ powder in the HP specimens, the 4HP group exhibited the greatest color change, with a statistically significant difference compared to the other groups (*p* < 0.05). This pronounced alteration in color could render the resin esthetically unacceptable, potentially limiting the application of high TiF_4_ concentrations in denture fabrication.

FTIR analysis confirmed the chemical interactions between TiF_4_ and the resin matrices. In the heat-polymerized groups, characteristic peaks at 1722 cm^−1^ (C=O stretching) and 1145 cm^−1^ (C–O–C stretching) were preserved across all concentrations, with only minor shifts, indicating weak interactions and structural stability of the resin. In contrast, the 3D-printed groups exhibited more pronounced changes, including shifts in the same functional groups and the emergence of a new peak at 605 cm^−1^, likely corresponding to Ti–F or Ti–O bonds. These findings suggest a strong and potentially uneven incorporation of TiF_4_ in the printed resin. Overall, FTIR spectra support that TiF_4_ interacts more consistently and forms a chemical bond in ND resins, while in HP resins, its distribution and bonding appeared less obvious. Such chemical interaction may have contributed to the higher variability in color change and performance of the ND groups compared to the HP groups, highlighting the need for further research into optimizing TiF_4_ incorporation in additive manufacturing workflows. The clinical significance of this study lies in the novel approach of modifying conventional dentures with TiF_4_, a material that has not been tested in the field of prosthodontics before. The findings indicate that incorporating TiF_4_ into heat-polymerized acrylic resin may enhance its antimicrobial properties, effectively inhibiting *C. albicans* biofilm development—a primary etiological factor in denture stomatitis. This antimicrobial modification holds promising clinical relevance, particularly in addressing the persistent challenge of biofilm accumulation on denture surfaces. The implications are especially pertinent for geriatric and immunocompromised populations, who are disproportionately affected by recurrent episodes of denture stomatitis. In these patients, chronic infections not only compromise oral function and comfort but may also contribute to systemic complications due to microbial translocation or sustained inflammatory responses. By reducing biofilm formation at the material interface, TiF_4_ treatment could limit the need for frequent prosthesis replacement, ultimately enhancing patient quality of life and reducing the financial and logistical burdens associated with long-term prosthodontic care. The modification could be applied after further studies on the effect of the TiF_4_ on the denture base materials. The TiF_4_ combination with the heat-polymerized PMMA did not have a negative impact on hardness and did not significantly increase the roughness compared to the unmodified material. However, the incorporation of 4% TiF_4_ into heat-polymerized resin significantly affected the resin color compared to lower tested concentrations, with no significant reduction in antifungal effect compared to the test groups. Additionally, this study underscores the importance of optimizing the mixing protocol for TiF_4_ in 3D-printed resins, which warrants further investigation to fully harness the advantages of additive manufacturing combined with antimicrobial agents. Moreover, exploring the strength and biocompatibility of this combination is also recommended for future research.

### Limitations and Recommendations

The study’s limitations include its in vitro nature, which does not fully replicate the oral environment. It is also suggested to test the long-term stability of the material and its effect on the denture bases. Additionally, the mixing method used for the printed resin is another limitation that could be improved in future studies. Furthermore, limited resin types and TiF_4_ concentrations were used. Therefore, further investigations with the addition of different concentrations of TiF_4_ to different resins and investigations simulating oral conditions are required.

## 5. Conclusions

The addition of TiF_4_ could reduce the *C. albicans* adhesion to heat-polymerized PMMA denture bases, and this decrease is concentration dependent with observed reductions of approximately 97.6% in the 1% of TiF_4_, 97.2% in the 2% of TiF_4_, 97.4% in the 3% of TiF_4_, and complete inhibition (100%) in the 4% of TiF_4_. The incorporation of TiF_4_ did not adversely affect PMMA hardness or significantly increase surface roughness. However, higher concentrations, particularly 4%, led to noticeable and unacceptable color changes. Therefore, while TiF_4_ showed promise as an antimicrobial additive, its concentration must be carefully controlled to maintain esthetic properties using lower concentrations.

For the 3D-printed resin, TiF_4_ did not significantly reduce *C. albicans* adhesion. However, TiF_4_ significantly increased hardness, with 2% TiF_4_ yielding the highest values. Color was also significantly affected, with 3% and 4% TiF_4_-treated groups showing the most color alteration. Regarding surface roughness, the addition of 1% and 2% significantly increased the roughness of the material.

This study highlights important considerations for researchers. The in vitro nature of the study does not fully replicate complex oral conditions, such as salivary flow, temperature changes, and microbial diversity. Additionally, only one type of 3D-printed resin and a limited range of TiF_4_ concentrations were evaluated. These limitations underscore the need for future investigations involving various denture base materials, broader concentration ranges, and in vivo testing to validate clinical applicability. Researchers must also consider long-term effects, biocompatibility, and the impact of TiF_4_ on other physical and mechanical properties of the materials.

The study findings suggest that TiF_4_’s effects on antifungal properties, roughness, and mechanical characteristics might be influenced by the type of resin material used and TiF_4_ concentration.

## Figures and Tables

**Table 1:** Material specifications of heat-polymerized PMMA, 3D-printed resin, and TiF_4_.

Material	Brand Name and Manufacturer Details	Composition
Heat-polymerized PMMA (HP)	HP, Probase Hot, Ivoclar Vivadent, Lichtenstein	Powder: Polymethyl methacrylate, softening agent, benzoyl peroxide, pigments Liquid: Methyl methacrylate, dimethacrylate (linking agent), catalyst
3D-printed resin	ND, NextDent Denture 3D+, 3D systems, Vertex Dental B.V., Soesterberg, Netherland	Ethoxylated bisphenol A dimethacrylate, 7,7,9(or 7,9,9)-trimethyl-4,13-dioxo-3,14-dioxa-5,12-diazahexadecane-1,16-diyl bismethacrylate, 2-hydroxyethyl methacrylate, Silicon dioxide, diphenyl(2,4,6-trimethylbenzoyl)phosphine oxide, Titanium dioxide
Titanium tetrafluoride powder (TiF_4_)	Aldrich Chemical Company, Milwaukee, WI, USA	Four atoms of fluoride per titanium atom

**Table 2:** Mean values (SD) and the significance of *C. albicans* colony, surface roughness, hardness, and color change considering the effect of different concentrations of TiF_4_ for heat-polymerized groups.

Properties	Mean (SD)					*p*
	HP (Control)	1HP	2HP	3HP	4HP	
*C. albicans (CFU)*	10,233.3 (787.0) ^a,b,c,d^	247.8 (63.2) ^a^	284.4 (42.4) ^b^	271.1 (50.4) ^c^	0 ^d^	<0.001 *
**Surface Roughness (Ra**, µm**)**	0.66 (0.3)	1.04 (0.01)	0.33 (0.03)	1.11 (0.09)	0.64 (0.03)	0.062
**Hardness (VHN)**	28.0 (3.7)	28.0 (3.7)	27.5 (3.5)	29.5 (3.3)	27.7 (1.9)	0.169
**Color Change (ΔE_00_)**	-----	8.25 (1.35) ^a^	7.19 (1.6) ^b^	9.02 (1.59) ^c^	11.78 (1.38) ^a,b,c^	<0.001 *

* Statistically significant at the 0.05 level of significance. The same small letters in each row show significant differences between the pairs.

**Table 3:** Mean values (SD) and the significance of *C. albicans* colony, surface roughness, hardness, and color change considering the effect of different concentrations of TiF_4_ for 3D-printed groups.

Properties	Mean (SD)					*p*
	ND (Control)	1ND	2ND	3ND	4ND	
*C. albicans (CFU)*	2566.7 (221.1)	2780 (272.4)	2044.4 (870.5)	2188.9 (108.4)	2817.8 (118.4)	0.913
**Surface Roughness (Ra**, µm**)**	1.23 (0.05) ^a,b^	2.26 (0.05) ^a,c^	2.51 (0.04) ^b,d,e^	1.37 (0.04) ^d,f^	0.39 (0.01) ^c,e,f^	<0.001 *
**Hardness (VHN)**	24.24 (1.8) ^a^	27.4 (3.8) ^b^	34.9 (1.8) ^a,b,c,d^	26.3 (1.6) ^c^	26.7 (1.8) ^d^	0.002 *
**Color Change (ΔE_00_)**	-----	3.91 (0.68) ^a,b,c^	2.05 (0.36) ^a,d,e^	5.42 (0.5) ^b,d,f^	7.89 (0.53) ^c,e,f^	<0.001 *

* Statistically significant at the 0.05 level of significance. The same small letters in each row show significant differences between the pairs.

## Data Availability

The original contributions presented in this study are included in the article. Further inquiries can be directed to the corresponding authors.
